# AXIN-AMPK signaling: Implications for healthy aging

**DOI:** 10.12688/f1000research.74220.1

**Published:** 2021-12-08

**Authors:** Avijit Mallick, Bhagwati P. Gupta

**Affiliations:** 1 Department of Biology, McMaster University, Hamilton, Ontario, L8S4K1, Canada

**Keywords:** Axin, AMPK, muscle, aging, C. elegans, LKB1, AAK-2, PRY-1

## Abstract

The energy sensor AMP kinase (AMPK) and the master scaffolding protein, AXIN, are two major regulators of biological processes in metazoans. AXIN-dependent regulation of AMPK activation plays a crucial role in maintaining metabolic homeostasis during glucose-deprived and energy-stressed conditions. The two proteins are also required for muscle function. While studies have refined our knowledge of various cellular events that promote the formation of AXIN-AMPK complexes and the involvement of effector proteins, more work is needed to understand precisely how the pathway is regulated in response to various forms of stress. In this review, we discuss recent data on AXIN and AMPK interaction and its role in physiological changes leading to improved muscle health and an extension of lifespan. We argue that AXIN-AMPK signaling plays an essential role in maintaining muscle function and manipulating the pathway in a tissue-specific manner could delay muscle aging. Therefore, research on understanding the factors that regulate AXIN-AMPK signaling holds the potential for developing novel therapeutics to slow down or revert the age-associated decline in muscle function, thereby extending the healthspan of animals.

## Introduction

With aging, there is a decline in skeletal muscle mass and function. Aging muscle undergoes a shift in the balance between myogenic potential and fibrogenic activity that leads to reduced capacity of the muscle to repair and regenerate. ^1^ Studies have shown that age-associated decline in muscle function is multifactorial and affected by genetic and environmental factors. While many genes have been identified that contribute to muscle development and function, their mechanisms of action are not well understood.

This review discusses a novel signaling network involving AXIN and AMP-activated protein kinase (AMPK) in maintaining muscle health that offers a new perspective on promoting healthy aging. Both these proteins are conserved in metazoans. AXIN is an established scaffolding protein that acts to integrate inputs from multiple signaling molecules, leading to the regulation of downstream effectors. ^2^ AMPK plays a crucial role in sensing intracellular energy levels and keeping a balance between cellular metabolism and growth. ^3^

## AXIN-AMPK signaling

**Figure 1.**
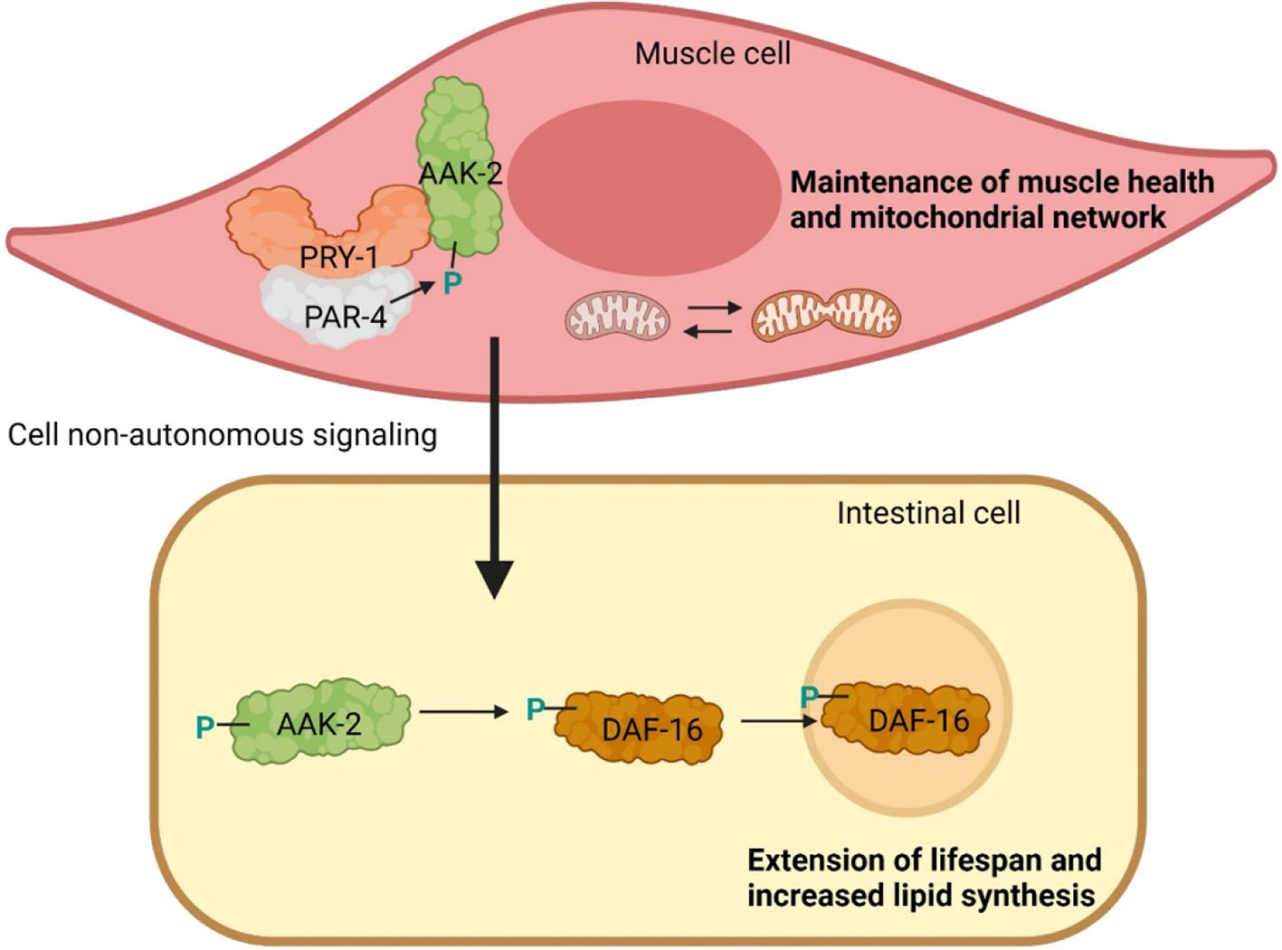
PRY-1/AXIN function in the muscle is necessary to maintain muscle health, mitochondrial biogenesis and longevity. Genetic and biochemical studies have shown that PRY-1/AXIN interacts with PAR-4/LKB1 and AAK-2/AMPK in muscles to promote AAK-2/AMPK phosphorylation. AAK-2 in turn activates DAF-16/FOXO cell non-autonomously in the intestine and promotes DAF-16/FOXO nuclear localization. Green colored P indicates activating phosphorylation.

**Figure 2.**
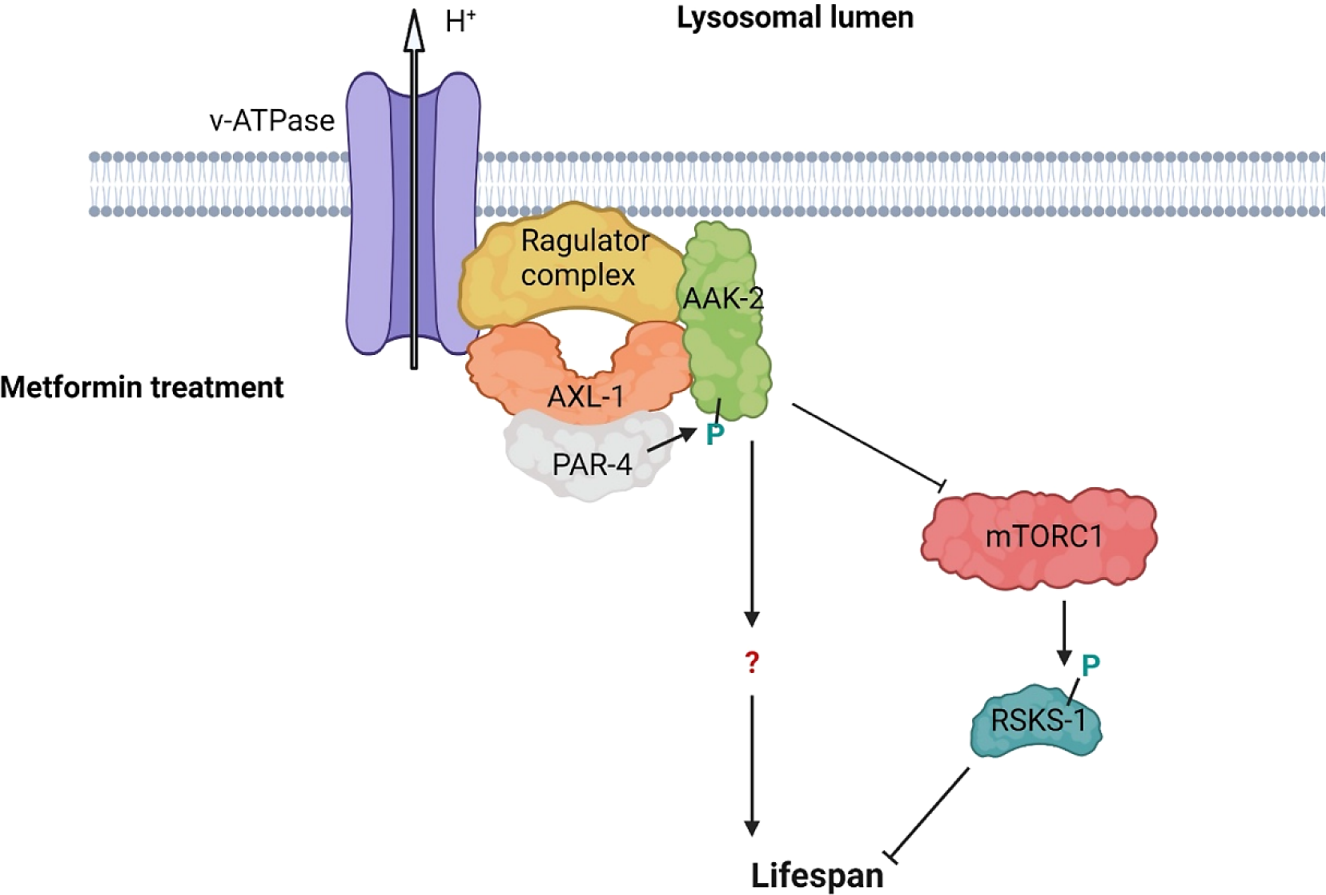
AXL-1/AXIN is required for metformin-mediated AAK-2/AMPK phosphorylation and longer lifespan in *C. elegans.* Metformin treatment induces formation of AXL-1/AXIN-based lysosomal VHA-3-LMTR-3-AXL-1/PAR-4-AAK-2 (v-ATPase-Ragulator-AXIN/LKB1-AMPK) complex that is required for AAK-2/AMPK activation. The effect of metformin is partially attained via inhibition of mTORC1, but other targets of the pathway remain unknown. Green colored P shows activating phosphorylation.

Recent findings from our lab and other published studies involving AXIN and its interacting partner AMPK provide a potential clue into the mechanism of muscle health maintenance. Work in the nematode *C. elegans* has revealed that the AXIN family member PRY-1 is necessary for animals’ normal motility and health, and its activated form promotes longevity by maintaining muscle mitochondrial homeostasis. ^4^ A similar function was previously ascribed to the AMPK catalytic subunit homolog AAK-2. ^5^^,^^6^ The genetic and biochemical experiments revealed that PRY-1 and AAK-2 work together, likely through protein-protein interaction, and PRY-1 is required for AAK-2-mediated beneficial effect on muscle health and lifespan ( Figure 1). The interaction between PRY-1 and AAK-2 is not a unique phenomenon, as other AXIN family members also interact with AMPK in different biological contexts. For example, another *C. elegans* AXIN homolog AXL-1 forms a complex with AAK-2 following metformin treatment. Here, AXL-1 is necessary for metformin-mediated lysosomal localization and activation of AAK-2 in a VHA-3-LMTR-3-PAR-4 (v-ATPase-Ragulator-LKB1) complex dependent manner ^7^ ( Figure 2).

The Axin-containing complexes are also reported in mammalian systems. Following metformin treatment and glucose deprivation, ^8^^–^^10^ the AXIN-based lysosomal pathway, consisting of v-ATPase-Ragulator complex (v-ATPase-Ragulator-AXIN/LKB1-AMPK), promotes AMPK phosphorylation by LKB1, leading to AMPK activation. In a separate study involving myotubes and mice gastrocnemius muscle tissue, exercise stimulated both AMPK and Rac1 while increasing the cellular levels of AXIN1. Accordingly, reducing the AXIN1 function blocked GTP loading of Rac1, AMPK activation, and glucose uptake in the exercising muscles. ^11^ Additionally, it was shown that muscle-specific knockout (KO) of the AXIN1-binding Ragulator subunit LAMTOR1 completely abolished treadmill exercise-stimulated AMPK activation in gastrocnemius muscle. ^10^ Together, these data demonstrate the crucial role of AXIN tethering in activating AMPK, which promotes muscle metabolism and benefits linked to exercise.

**Figure 3.**
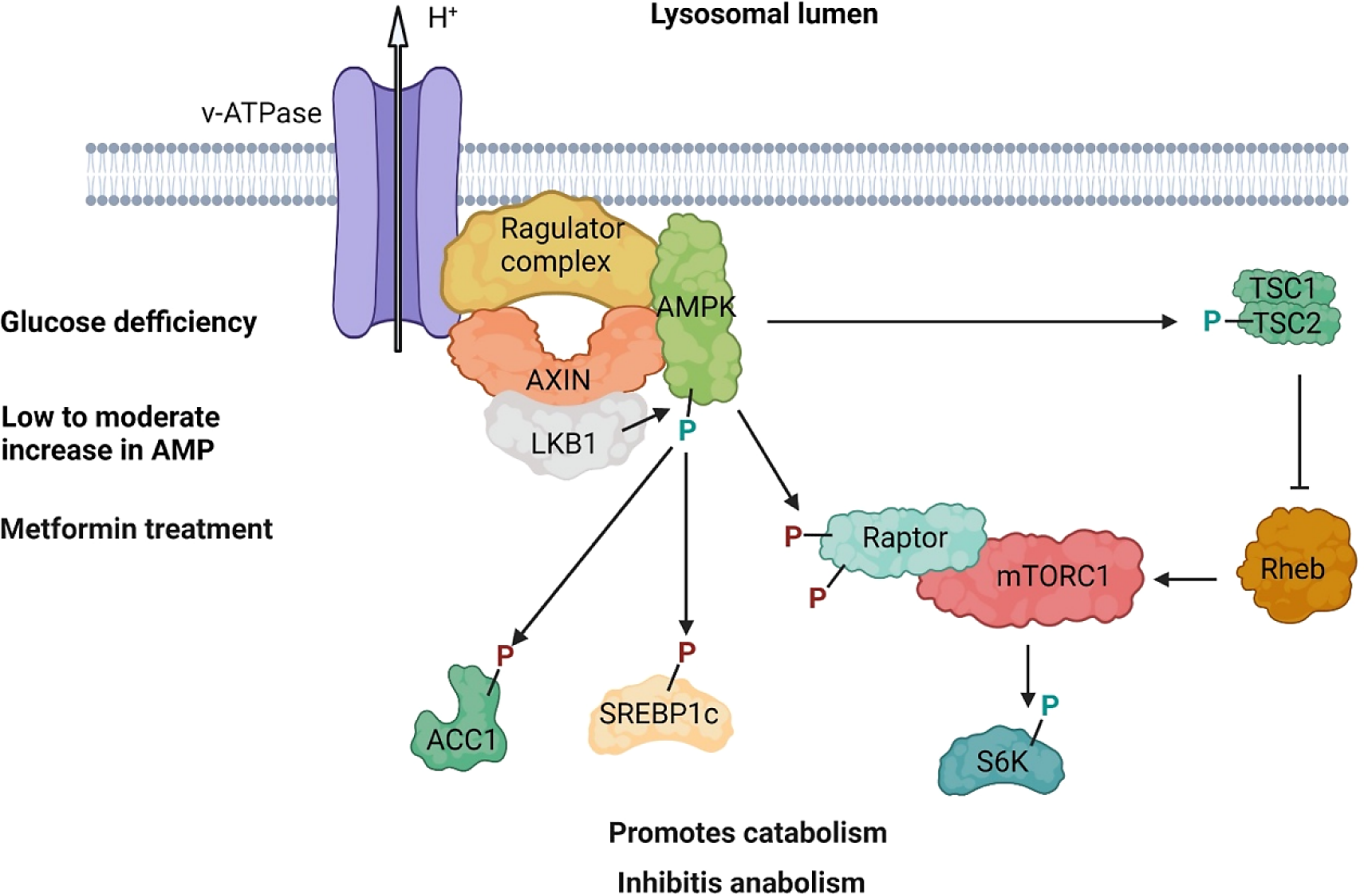
AXIN forms a lysosomal complex, v-ATPase-Ragulator-AXIN/LKB1-AMPK, that is crucial for AMPK activation and the maintenance of energy homeostasis during stress-inducing conditions. The complex is formed following glucose deprivation, low to moderate increases in AMP levels, and metformin treatment. Once activated, AXIN-AMPK signaling promotes catabolism and inhibits anabolism by phosphorylating downstream targets that include ACC1, SREBP1c, Raptor and TSC2. Green and purple colored P indicate activating and inhibitory phosphorylation, respectively.

Investigations of cellular mechanisms underlying AXIN and AMPK interaction have revealed a regulatory relationship that depends on AMP levels ^12^ ( Figure 3). While low glucose triggered AMP-dependent activation of AMPK through the AXIN-based lysosomal pathway, a modest increase in AMP resulted in AXIN-dependent activation of both lysosomal and cytosolic AMPK. Finally, extreme nutrient starvation or high AMP concentrations caused phosphorylation of AMPK independently of AXIN function. ^12^

Intriguingly, it was shown recently that skeletal muscle-specific AXIN1 knockout (AXIN1 imKO) mice are phenotypically normal and exhibited no impairment of AMPK regulation or glucose uptake. ^13^ Such a phenotype may be explained by redundancies between AXIN1 and its homolog AXIN2. Both proteins are expressed in skeletal muscles, and AXIN2 can functionally replace AXIN1 in regulating AMPK. ^12^^,^^14^ Moreover, AXIN2, a negative regulator of WNT signaling, appears to be essential for myogenesis, as increased WNT signaling in aged skeletal muscle promoted fibrogenesis, thereby accelerating aging. ^15^^–^^17^

Consistent with the role of AXIN in AMPK activation and myogenesis, AMPK is shown to be crucial for regulating skeletal muscle development, growth, and degradation. ^18^ In skeletal muscle, AMPK signaling has been linked to both acute and chronic exercise adaptations, in addition to a broad range of skeletal muscle disease states and ageing. ^19^^,^^20^ Together these data support the growing evidence that both AXIN and AMPK and their signaling cascade are crucial to maintaining healthy muscles and slowing organismal deterioration with aging.

## Downstream effectors of AXIN-AMPK signaling

Studies in mammalian models revealed that both metformin and glucose deprivation inhibit the mechanistic target of rapamycin complex 1 (mTORC1) activity, a master regulator of anabolic pathways. ^8^^,^^10^ Both these treatments cause the mTORC1 components, RAPTOR and mTOR, to dissociate from the v-ATPase-Ragulator and facilitate the formation of the v-ATPase-Ragulator-AXIN/LKB1-AMPK complex. Similarly, research in *C. elegans* has demonstrated that the VHA-3-LMTR-3-AXL-1/PAR-4-AAK-2 complex negatively regulates phosphorylation of the mTORC1 target S6 kinase B1 (S6K) homolog RSKS-1. ^7^ As the beneficial effects of AXIN-AMPK signaling in the *C. elegans* study were not directly attributed to mTORC1 inhibition, the authors suggested that the signaling cascade may utilize additional factors ^7^ ( Figure 2).

The downstream effectors of AXIN-AMPK have been reported in several other studies. Specifically, in a low glucose condition, the pathway phosphorylates proteins such as acetyl-CoA carboxylase (ACC1) and endoplasmic reticulum-localized sterol regulatory element-binding protein-1c (SREBP1c), thereby inhibiting fatty acid synthesis ^12^ ( Figure 3). Interestingly, in *C. elegans*, PRY-1 promotes transcription of SREBP1 homolog SBP-1 to regulate fatty acid synthesis ^4^^,^^21^; however, the precise mechanism of this regulatory relationship is unknown. Another effector of PRY-1 appears to be the CREB-regulated transcriptional coactivator (CRTC) homolog. ^22^ CRTC-1 is known to function downstream of AAK-2 and affects calcineurin-mediated lifespan and stress regulation in *C. elegans.* ^23^^,^^24^ While AMPK and calcineurin signaling in mammalian systems regulate CRTCs in an antagonistic manner, the involvement of Axin in this regulatory network remains to be determined. ^25^^–^^28^

Given that AMPK regulates many targets, it is expected that a subset may be co-regulated by AXIN. We recently reported that both *pry-1* and *aak-2* mutant transcriptomes significantly overlap with mutually up and downregulated genes. These common differentially expressed genes are associated with muscle structure development, muscle contraction, aging, and lipid metabolism. Moreover, we found that PRY-1-AAK-2 signaling functions in muscles leading to activation of AAK-2 in a cell-non-autonomous manner and phosphorylation and translocation of the FOXO transcription factor homolog DAF-16 into the intestinal cell nuclei ^4^ ( Figure 1). These results are supported by previous studies showing that activated DAF-16 is indispensable for muscle mitochondria homeostasis and lifespan extension. It is worth mentioning that FOXO3 is also phosphorylated by AMPK in the mammalian system; however, the involvement of AXIN in this process and the function of activated FOXO3 are unknown. ^29^

Unlike *C. elegans*, little is known about the role of AXIN and AMPK in regulating muscle health in another leading invertebrate model, namely the fruit fly *D. melanogaster.* Overexpression of *D-axin* in wing disc-associated myoblasts in larvae causes partial to complete loss of indirect flight muscles. ^30^ However, the precise role of *D-axin* and the involvement of AMPK and TORC1 in adult muscles is unknown. In terms of other processes, it has been reported that a hypomorphic allele of *D-axin* alters the expression of metabolic genes and is hypersensitive to metabolic stress induced by fasting. Such a phenotype depends on TORC1 activity and involves increased ROS production. ^31^

## Gaps in our knowledge

While much has been learned about Axin, AMPK, and their interactions, there are gaps in our understanding of the mechanisms regulating the complex formation, downstream effectors, and their role in maintaining muscle health. Some of the relevant questions are discussed below.

### Is AXIN expression beneficial for muscle health?

The existing data supports that AXIN function in the muscle is beneficial. AXIN2 is required for myogenesis and linked to muscle aging, whereas AXIN1 mediated signaling is necessary for glucose uptake in the exercising muscles. ^11^^,^^15^^,^^17^ Both AXIN1 and AXIN2 are expressed in the skeletal muscle. Research in *C. elegans* hints that muscle-specific overexpression of *pry-1* promotes mitochondrial network, muscle development, and muscle physiology. ^4^ Whether such a role of Axin is conserved in higher eukaryotes is unknown.

### Are AXIN1 and AXIN2 redundant in activating AMPK?

While AXIN1 and AXIN2 possess similar domains, they show differences in their regulation and expression pattern (subcellular localization and cell type-specific expression). ^14^^,^^32^ Additionally, AXIN2 is required for muscle development. Interestingly, exercise-induced glucose uptake requires AXIN1 in skeletal muscles. While it remains to be seen whether AXIN2 plays a redundant role in this process and regulates AMPK, Li *et al*. ^13^ reported no change in AMPK activation following AXIN1 imKO in the skeletal muscle. Furthermore, Zong *et al*. ^12^ showed that AXIN2 could substitute AXIN1 in forming a complex between LKB1 and AMPK.

In *C. elegans*, PRY-1 and AXL-1 possess the characteristic domains for the AXIN family of proteins ^2^ and negatively regulate WNT signaling. ^33^^,^^34^ It has been shown that AXL-1 functions redundantly with PRY-1 to regulate the WNT effector protein BAR-1/β-catenin during the formation of the vulva and migration of Q neuroblast. However, both AXINs are functionally not equivalent and play roles independently to control specific processes. For example, PRY-1 is necessary for lipid metabolism, healthspan, lifespan, and seam cell development, whereas AXL-1 regulates excretory cell development. ^4^^,^^21^^,^^33^^–^^36^ Recent experiments from our lab also highlight functional differences between the two Axin proteins. While PRY-1 and AXL-1 are necessary for metformin-induced lifespan extension, ^7^ only PRY-1 is required for glucose deprivation mediated longevity in *C. elegans* (Mallick *et al*., unpublished). These same treatments, i.e., metformin and glucose deprivation, are known to extend the lifespan in an AAK-2-dependent manner. ^37^^,^^38^ Overall, these studies demonstrate that AXIN homologs in every system have shared as well as unique functions. However, whether these proteins can redundantly activate AMPK remains to be investigated.

### What factors limit AXIN-AMPK signaling?

Recent reports demonstrate that the lysosomal AXIN-AMPK signaling can be activated by glucose deprivation independently of AMP/ATP ratios. However, the medium-to-high elevation of AMP extends the activation of both cytosolic and lysosomal AMPK, which is also dependent on AXIN1. ^12^ By contrast, very high AMP levels phosphorylate AMPK in a manner that does not involve AXIN1 and probably occurs via a conformational change in AMPK. Whether AXIN-dependent activation of AMPK also requires a similar change in AMPK conformation is unclear. Furthermore, it is unknown how glucose levels facilitate the complex formation and differential activation of AMPK by LKB1.

Several other factors may also limit AXIN and AMPK mediated signaling. One of these is post-translational modification. AXIN activity is known to be regulated by phosphorylation. ^2^^,^^39^^,^^40^ Another could be subcellular localization. While the AXIN-AMPK complex is localized to lysosomes and cytoplasm, the changes in their activities in response to external stimuli are poorly understood. ^10^^,^^12^ Both factors are broadly expressed and in overlapping domains; however, whether their interactions are global or restricted to specific tissues remains to be determined. In this regard, it is worth mentioning that AMPK functions cell non-autonomously in *C. elegans,* ^24^ and we have reported that the protein is needed in both muscles and intestine to mediate beneficial effects of constitutive expression of AXIN in muscles. ^4^

### What are the effectors of AXIN-AMPK signaling?

Given that AMPK is involved in many different processes and regulates many downstream targets, one might expect that AXIN-AMPK interaction co-regulates a subset of the targets. In support of this, a recent paper suggests that AXIN-AMPK signaling phosphorylates targets that are different from ATP/AMP-dependent AMPK signaling. ^12^ As mentioned above, our analysis of *C. elegans pry-1* and *aak-2* transcriptomes has revealed many overlapping genes that are differentially expressed. However, more work is needed to identify and validate common targets of AXIN-AMPK signaling that are involved in maintaining muscle health in different systems. Identification of such target genes could lead to a better understanding of molecular mechanisms underlying the signaling network and the development of diagnostic markers and therapeutic interventions to promote muscle health.

## New research directions

We envisage several exciting research avenues involving AXIN-AMPK signaling. While substantial knowledge has been gained in terms of processes that each one participates in and mechanisms underlying their function, little is known how the interactions between the two proteins are regulated, leading to changes in the expression of target genes that carry out various roles. Below are some of the potential research directions to address the questions in the previous section.

While it has been shown that the AXIN homologs in both *C. elegans* (PRY-1 and AXL-1) and mammalian systems (AXIN1 and AXIN2) can activate AMPK, ^4^^,^^7^^,^^9^^,^^12^ the redundancies between the homologs and their tissue-specific interactions with AMPK are unknown. Moreover, the differences in lifespan and lipid metabolism phenotypes between the two AXIN mutants in *C. elegans* raise the question of functional equivalency regarding AMPK activation in physiological conditions. Future research along these lines should refine our understanding of AXIN-AMPK signaling and its conservation in eukaryotes.

Depending on the context, signaling pathways may utilize different mechanisms to regulate their responses. In this regard, research in the following areas should improve our understanding of the regulatory mechanism of AXIN-AMPK signaling. First, whether a conformational change in AMPK following AXIN binding occurs similar to the AMP-dependent mechanism. Second, the role of post-translational modification of AXIN in activating AMPK. Third, identifying a specific region of the multidomain AXIN protein required for AMPK interaction that, in turn, may uncover potential competitors to modulate the signaling. And, finally, the discovery of factors affecting subcellular localizations of both AXIN and AMPK and, in turn, their interactions.

Other modes of regulation of AXIN-AMPK signaling may include spatial and temporal changes in AXIN expression. AXIN is not only a negative regulator but also a downstream target of the WNT signaling. ^21^^,^^41^ Consistent with this, PRY-1/AXIN is required for MOM-2/WNT mediated lifespan regulation, ^4^ and MOM-2 is expressed in the body wall muscles of *C. elegans.* It remains to be explored whether AXIN function in muscles is regulated in a WNT-dependent manner in eukaryotes.

Research from our group has shown that overexpression of PRY-1/AXIN in *C. elegans* extends the lifespan and improves muscle health in older adults. Whether forced expression of mammalian AXIN in muscles may also promote the healthspan of animals by activating AXIN-AMPK signaling requires investigation. In line with this, expression analysis of AXIN1 and AXIN2 in old adults and patients with a muscle disease should prove valuable.

As mentioned above, AXIN and AMPK are crucial for muscle development and physiology. Furthermore, exercise promotes the activation of AMPK in an AXIN-dependent manner. Given that exercise promotes muscle health and delays aging, ^42^^–^^44^ it is conceivable that AXIN and AMPK are involved in this process. More work is needed to understand the role of AXIN-AMPK signaling in exercise-mediated benefits.

## Conclusion

AXIN family of scaffolding proteins control a wide array of cellular processes by recruiting multiple factors and forming protein complexes. One of the interactors of AXIN is the well-known energy sensor AMPK. AMPK functions as a nexus between energy conservation and aging, and perturbations of its function lead to various age-related pathologies. AXIN-AMPK signaling promotes muscle health and delays age-associated deterioration. Future studies on the pathway, its interacting proteins, and tissue-specific effectors hold promise to uncover key candidates that may be targeted in the future to delay age-associated muscle degeneration and improve muscle health during aging.

## Data availability

No data are associated with this article.
