# Supplementary material for: AXIN-AMPK signaling: Implications for healthy aging
Source: F1000Res. 2021 Dec 8;10:1259. [Version 1] doi: 10.12688/f1000research.74220.1 (PMC8764564; doi:10.12688/f1000research.74220.1)
Supplement: Peer review history

# Peer review: Reviewer response for version 1

**DOI:** 10.5256/f1000research.77950.r102286

**Published:** 2022-01-17

**Authors:** Stefan Taubert

**Affiliations:** 1 Department of Medical Genetics, University of British Columbia, Vancouver, BC, Canada

This review/opinion article considers the roles of the two AXIN type proteins in AMPK signaling, focusing on muscle function and primarily on research done in C. elegans. The article nevertheless balances well what is known from different species, and highlights several areas for future investigation. The article is well written and the figures are useful.

 There are no major issues, as the discussion is balanced and inclusive of knowledge gained from various systems. It would have perhaps been useful to compare what is known about the two different AXIN proteins in a table (interacting proteins, expression, location, etc) to highlight differences in an easily accessible resource.

Is the topic of the opinion article discussed accurately in the context of the current literature?

Yes

Are arguments sufficiently supported by evidence from the published literature?

Yes

Are all factual statements correct and adequately supported by citations?

Yes

Are the conclusions drawn balanced and justified on the basis of the presented arguments?

Yes

Reviewer Expertise:

C. elegans, transcription, metabolism, stress response, longevity,

I confirm that I have read this submission and believe that I have an appropriate level of expertise to confirm that it is of an acceptable scientific standard.

# Peer review: Reviewer response for version 1

**DOI:** 10.5256/f1000research.77950.r108092

**Published:** 2022-01-06

**Authors:** Anindya Ghosh Roy

**Affiliations:** 1 Department of Cellular and Molecular Neuroscience, Nationa Brain Research Centre, Gurgaon, Haryana, India

The ‘Opinion article’ by Mallick and Gupta focusses on the in vivo function of AXIN-AMPK axis in muscle homeostasis and organismal health/aging. AMPK has long been known to regulate energy production in the context of change of ATP level due to stress, physical exercise etc. It has multiple functions and it is regulated by various means. The mode of its activation is different in response to differential levels of ATP depletion. Therefore figuring out the downstream effectors and biological effect in the context of differential levels of AMPK activation has been challenging.

 There are various evidences in last few years that a scaffolding protein AXIN binds to AMPK and regulates the AMPK signaling positively. These evidences come from studying multiple model organisms including mice, flies and worms. The more recent finding from this group that AXIN and AMPK interaction plays an important role in delaying the muscle aging and organism level aging in *C elegans* model is very interesting (Mallick et al 2020). Moreover, the AMPK signaling helps the crosstalk between muscle and intestine to coordinate the aging process. Therefore, focusing on this topic to write this short review article is very timely and appropriate.

 I have a few suggestions on the organization of this article.

1. First the authors should avoid referring to their article actively with phrases such as  “study from our lab/group”. They may mention “recent study” instead.
2. I suggest that they first discuss the in vivo findings on AXIN-AMKP signaling in other organisms such as mice and flies in muscle health, ATP homeostasis etc. They can have a sub-heading for this. Then they can bring a separate sub-titled section, which describes the findings using worm model focusing their recent findings. Essentially, I am suggesting that they break the section titled ‘AXIN-AMPK signaling’ in two parts as suggested above. This will allow them to discuss the key interesting hypothesis coming out from their work.
3. One point to highlight in the future direction/question is how AXIN-AMPK signaling in muscles regulates the intestinal regulation of DAF-16. They can have a sub-title on this. Please suggest a few possibilities.
4. Another point is to propose some research questions on how AXIN intersects between Wnt and AMPK signaling. What are the interesting questions in this direction that can be solved using the worm model?

Is the topic of the opinion article discussed accurately in the context of the current literature?

Yes

Are arguments sufficiently supported by evidence from the published literature?

Yes

Are all factual statements correct and adequately supported by citations?

Yes

Are the conclusions drawn balanced and justified on the basis of the presented arguments?

Yes

Reviewer Expertise:

Genetics, C elegans, neural circuit development, neuronal regeneration, aging

I confirm that I have read this submission and believe that I have an appropriate level of expertise to confirm that it is of an acceptable scientific standard, however I have significant reservations, as outlined above.

# Peer review: Reviewer response for version 1

**DOI:** 10.5256/f1000research.77950.r102289

**Published:** 2022-01-05

**Authors:** Jonathan S. Oakhill

**Affiliations:** 1 University of Melbourne, Melbourne, Vic, Australia

The authors provide a largely balanced overview of current understanding regarding co-ordination between the energy sensor AMPK and well-defined scaffold protein AXIN1 to promote healthy aging, and succinctly outline knowledge gaps and future directions. A major question (for this protein biochemist at least) remains whether this occurs via direct interaction as the text and figures here suggest. Albeit an opinion piece, I think there is a degree of extrapolation and overstatement. For example, I could not find evidence in ref 7 to support the statement “...another C. elegans AXIN homolog AXL-1 forms a complex with AAK-2 following metformin treatment” on p3. Also AXIN2 redundancy has been demonstrated at the functional level, not structural, as stated on p6.

 The AXIN1/AMPK complex was reported in 2013 but has not been independently verified as far as I’m aware. It is not a stretch to say the model has caused controversy in the field – mainly created by difficulty in replicating the primary co-IP and this should be given due accord. The authors published work using C. elegans notes that a physical interaction requires further examination.

 I suggest the article would benefit from clearly distinguishing ‘function’ from ‘interaction’. I also recommend elaboration on the nature of the molecular interaction between AMPK and AXIN1, for which biochemical data are reported (eg Zhang et al, 2013 Fig 5). Authors could comment on requirement for AMPK b-subunit myristoylation for formation of the axin/AMPK complex in vitro. The non-myristoylatable mutant b2-G2A failed to localise to lysosomes following glucose starvation. Likewise requirement for the AXIN1 region between residues 507-731, which is indispensable for stabilisation of the complex in vitro but is outside of conserved AXIN1/2 domains.

 Please expand on what is known of the major AXIN regulatory post translational modifications.

Is the topic of the opinion article discussed accurately in the context of the current literature?

Partly

Are arguments sufficiently supported by evidence from the published literature?

Partly

Are all factual statements correct and adequately supported by citations?

Partly

Are the conclusions drawn balanced and justified on the basis of the presented arguments?

Yes

Reviewer Expertise:

Metabolic signaling pathways; structural biology; cell biology

I confirm that I have read this submission and believe that I have an appropriate level of expertise to confirm that it is of an acceptable scientific standard, however I have significant reservations, as outlined above.
